# Exposure to combustion derived particulate matter exacerbates influenza infection in neonatal mice by inhibiting IL22 production

**DOI:** 10.1186/s12989-021-00438-7

**Published:** 2021-12-14

**Authors:** Avinash Kumar, Vivek S. Patel, Jeffrey N. Harding, Dahui You, Stephania A. Cormier

**Affiliations:** 1 Department of Biological Sciences, Louisiana State University, Baton Rouge, LA USA; 2 Pennington Biomedical Research Center, 6400 Perkins Road, Baton Rouge, LA USA; 3 Department of Pediatrics, University of Tennessee Health Sciences Center, Memphis, TN USA

**Keywords:** Particulate matter, Influenza, IL-22, Microbiome, Tryptophan metabolites

## Abstract

**Background:**

Particulate matter (PM) containing environmentally persistent free radicals (EPFRs) are formed during various combustion processes, including the thermal remediation of hazardous wastes. Exposure to PM adversely affects respiratory health in infants and is associated with increased morbidity and mortality due to acute lower respiratory tract infections. We previously reported that early-life exposure to PM damages the lung epithelium and suppresses immune responses to influenza virus (Flu) infection, thereby enhancing Flu severity. Interleukin 22 (IL22) is important in resolving lung injury following Flu infection. In the current study, we determined the effects of PM exposure on pulmonary IL22 responses using our neonatal mouse model of Flu infection.

**Results:**

Exposure to PM resulted in an immediate (0.5–1-day post-exposure; dpe) increase in IL22 expression in the lungs of C57BL/6 neonatal mice; however, this IL22 expression was not maintained and failed to increase with either continued exposure to PM or subsequent Flu infection of PM-exposed mice. This contrasts with increased IL22 expression in age-matched mice exposed to vehicle and Flu infected. Activation of the aryl hydrocarbon receptor (AhR), which mediates the induction and release of IL22 from immune cells, was also transiently increased with PM exposure. The microbiome plays a major role in maintaining epithelial integrity and immune responses by producing various metabolites that act as ligands for AhR. Exposure to PM induced lung microbiota dysbiosis and altered the levels of indole, a microbial metabolite. Treatment with recombinant IL22 or indole-3-carboxaldehyde (I3A) prevented PM associated lung injury. In addition, I3A treatment also protected against increased mortality in Flu-infected mice exposed to PMs.

**Conclusions:**

Together, these data suggest that exposure to PMs results in failure to sustain IL22 levels and an inability to induce IL22 upon Flu infection. Insufficient levels of IL22 may be responsible for aberrant epithelial repair and immune responses, leading to increased Flu severity in areas of high PM.

## Introduction

Ambient air pollution is a rising public health issue, which is responsible for approximately 3.1 million premature deaths per year worldwide [1]. One of the main contaminants contributing to atmospheric air pollution is combustion-derived particulate matter (PM) [2]. PM is emitted during industrial combustion processes, vehicle and household fuel combustion, cigarette (and e-cigarette) smoking, wildfires, and the thermal remediation of hazardous wastes [3–5]. In addition to their role in increasing oxidative stress and inflammation [6, 7] exposure to PM is also associated with the development of cardiovascular diseases [7–10], diabetes [11, 12], cancer [12, 13] and neurological diseases [14, 15]. Most importantly, exposure to PM has frequently been associated with aggravated respiratory diseases such as lower respiratory tract infections and asthma, especially in infants and children [16–21]. Although there is plenty of evidence regarding the deleterious effects of PM exposure in infants and children [22–24], very few studies have explored the underlying mechanisms in this susceptible population. Previously, we reported that exposure to PM in neonatal mice damaged the lung epithelial layer, induced airway remodeling and increased the severity of influenza infection [25, 26]. Further studies are required to identify the molecular mechanisms underlying PM-exacerbated influenza infection in infants.

The maintenance of barrier integrity and function is crucial at the exposed surfaces of mammalian system such as lung epithelial layer in response to environmental stimulants. Interleukin-22 (IL22), an effector cytokine of IL-10 family of cytokines, plays an important role in epithelial layer repair and maintenance and epithelial barrier integrity [27, 28]. Using the *Klebsiella pneumoniae* model of pneumonia, Aujla et al. demonstrated the host defense role of IL22 in the lung [29]. In addition to their role in epithelial barrier maintenance, IL22 has been involved in balancing epithelial cell permeability and growth, production of antimicrobial proteins, mucus, and also, compliment production [27, 30]. The protective role of IL22 during fungal and bacterial infection is well documented [29, 31]; however, there are controversial reports on the importance of IL22 functions in influenza infection. Initial reports demonstrated only a minor role for IL22 during H1N1 influenza A virus infection in mice and as measured by influenza related morbidity and mortality [32]. Additionally, there was no effect on lung function or respiratory tissue remodeling [33] or viral load and survival following lethal influenza infection and CD8 T cell responses [34]. Paradoxically, this latter paper further demonstrated that IL22 was important in protecting against pulmonary inflammation during sublethal influenza infection and that it was critical in providing resistance to secondary bacterial infections [34]. The role of IL22 in PM-exacerbated influenza severity has not been studied.

The development and function of immune system is highly dependent on commensal microbiota [35]. The microbiome plays an important role in development and training of major components of the host’s adaptive and innate immune system [35, 36]. The microbiome also regulates several physiological functions, such as drug metabolism, nutrient metabolism and immune responses to antigens [37]. The intestinal microbial community increases immunity by driving the production of IL22 by ILC3s thus providing innate immune defense at mucosal surfaces [38]. Similarly experiments with germ free mice demonstrated impaired production of IL22 suggesting the requirement of commensal microbiota [39]. Gut microbiota also help maintain intestinal epithelial integrity by producing different metabolites. In a recent study it was demonstrated that short chain fatty acids derived from gut microbiota induce IL22 in ILCs and CD4 + cells and are important for maintaining intestinal homeostasis [40]. Some of the amino acid metabolites, e.g., tryptophan metabolites, that are produced by commensal microbiota, act as a substrate for the aryl hydrocarbon receptor (AhR), which in turn induces immune cells to produce IL22 [41, 42]. In fact, administration of IL22 to AhR-/- mice, which have reduced IL22 production, protects the host from *Citrobacter rodentium* infection [43]. Thus, a healthy and diverse microbiome is important for maintenance of epithelial barrier and induction of immune responses against various invading pathogens. Recent reports indicate that exposure to PM and other air pollutants alter the lung microbiome [44]. However, there are no studies that have explored the effects of PM exposure on lung microbiome and resistance to influenza infection. In this study, we determined the effects of PM exposure on IL22 responses following influenza infection. Further, we determined if PM exposure affects the lung microbiota and consequent AhR activation.

## Results

### Long-term exposure to PM suppresses IL22 expression following influenza infection by altering AhR activation

**Fig. 1:**
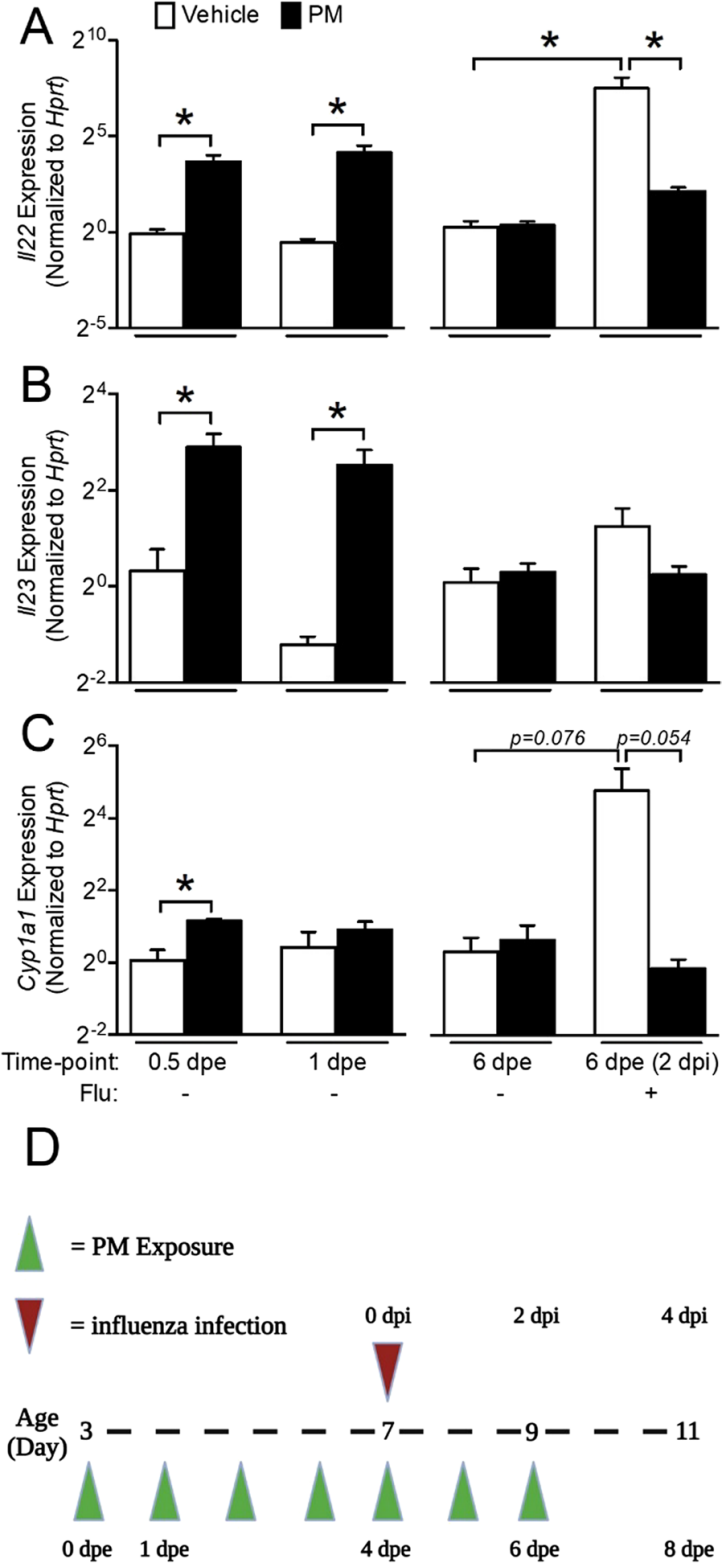
Continuous exposure to PM fails to sustain Il22 expression and inhibits Il22 expression after influenza infection in the lungs. Neonatal mice (age: 3 days) were exposed to aerosolized PM and infected with influenza or sham at 4 dpe. Lungs were collected at 0.5, 1 and 6 dpe and at 2 dpi. **A** Il22, **B** Il23 and **C** Cyp1a1 expression were measured using qRT-PCR. Data are normalized to vehicle-exposed 0.5 dpe group and vehicle-exposed non-infected group at 2 dpi. n = 3–5/group, *p < 0.05. **D** Schematic of the exposure, infection and sampling timepoints

IL22 helps maintain the lung epithelial layer by inducing repair mechanisms following sterile or infectious injuries [27, 28]. We determined the expression of IL22 at different times following PM exposure and influenza infection in lungs. IL22 expression was significantly higher in PM-exposed mice at 0.5 and 1 dpe (without influenza infection) compared to vehicle-exposed mice. There was no difference in the expression at 6 dpe between the groups (Fig. 1A). These results indicate that exposure to PM initially induces IL22 expression in the lungs, but this increase is not sustained with multiple exposures (i.e., at 6 dpe). We further measured IL22 expression in PM-exposed mice infected with influenza virus. The vehicle-exposed influenza infected mice showed a significant increase in IL22 expression as compared to vehicle-exposed sham infected mice. On the contrary, the PM-exposed influenza infected mice did not show such an increase in lung IL22 expression. Similar expression trends were observed for IL23, an IL22 inducing cytokine. PM-exposed mice failed to show an increase in IL23 expression following influenza infection (Fig. 1B).

AhR binds to a variety of exogenous and endogenous ligands and controls the induction of IL22 in immune cells, and thus helps in the repair of lung epithelium. We determined the expression of Cyp1a1, a marker for AhR activation, in PM-exposed and influenza-infected mice. Similar to IL22 and IL23, Cyp1a1 expression was significantly increased at 0.5 dpe, but this increase had abated by 1 dpe and later at 6 dpe (Fig. 1C). There was no difference in the expression between sham-infected vehicle-exposed and PM-exposed mice at 6 dpe. Following influenza infection, Cyp1a1 expression increased significantly in vehicle-exposed mice but failed to increase in PM-exposed mice. Together, these results suggest an association between suppressed IL22 expression and altered AhR activation and indicate that failure of AhR activation may have led to suppressed IL22 expression following influenza infection.

### Long-term exposure to PM suppresses induction of IL22 + immune cells following influenza infection

**Fig. 2:**
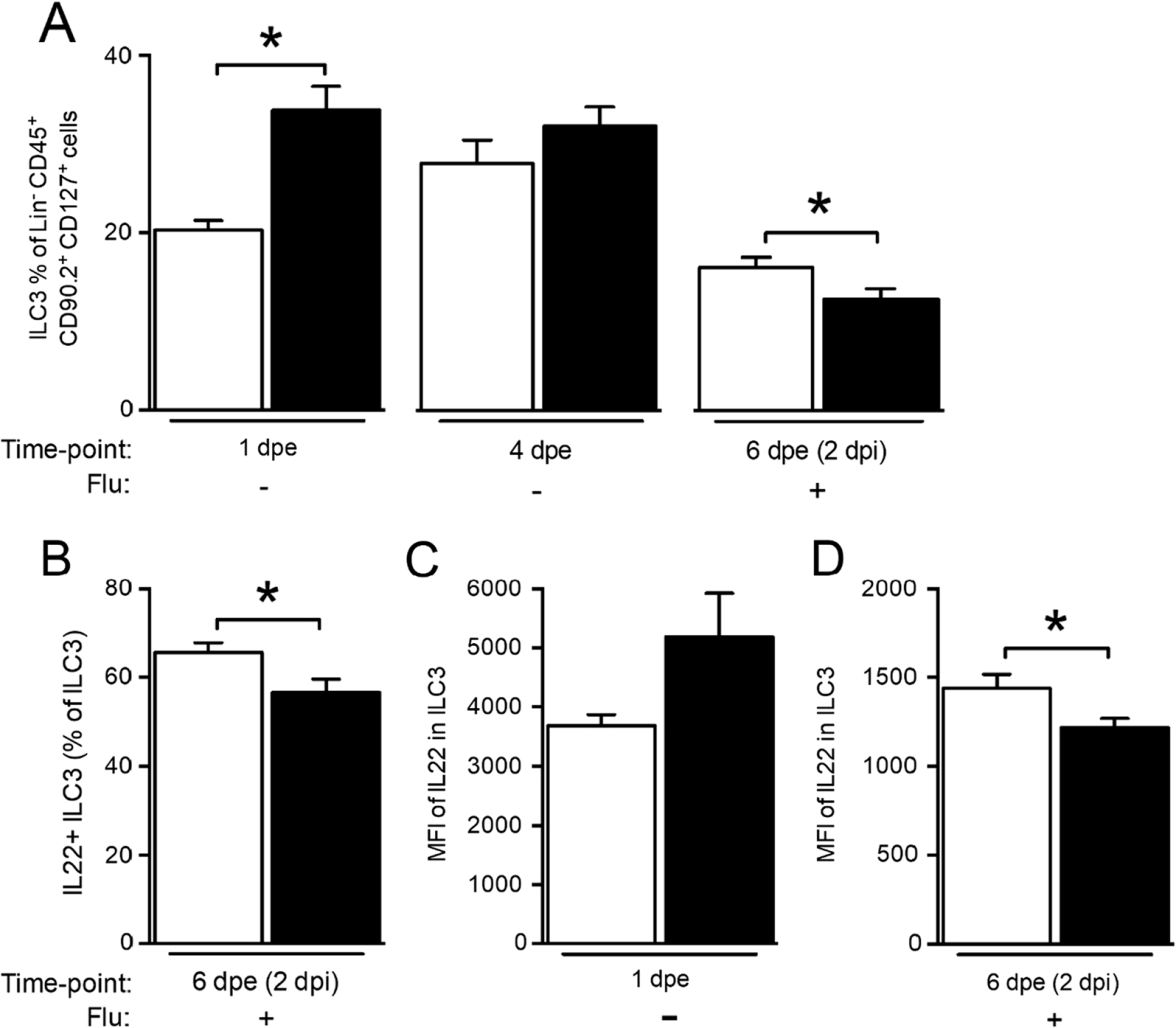
ILC3 cells increase immediately following exposure to CDPM. Neonatal mice (age: 3 days) were exposed to aerosolized PM and infected with influenza or sham infected at 4 dpe. Lungs were collected at 1 dpe, 4 dpe or 6 dpe (2 dpi) (**A**–**D**). Lung cells were stained for surface and intracellular markers. Target cell population and Il22 expression were analyzed using flow cytometry. n = 4–8/group, *p < 0.05

Type 3 innate lymphoid cells (ILC3) play a major role in maintenance and repair of gut epithelium and are one of the major sources of IL22 [45]. We sought to determine the effect of long-term PM exposure on lung ILC3. After initial exposure to PM, the percent of ILC3 significantly increased at 1 dpe, but such increase was not seen later at 4 dpe. Upon infection with influenza, the percent of ILC3 increased in the vehicle-exposed mice, but not in PM-exposed mice (Fig. 2A, B). In addition, the MFI of intracellular IL22 in ILC3 was significantly higher at 1 dpe, but failed to increase later at 2 dpi in PM-exposed mice (Fig. 2C, D). The numbers of ILC3 and intracellular levels of IL22 correlated with IL22 expression in the lungs of PM-exposed mice.

### Long-term PM exposure induces lung microbiota dysbiosis and alters microbial metabolites

**Fig. 3:**
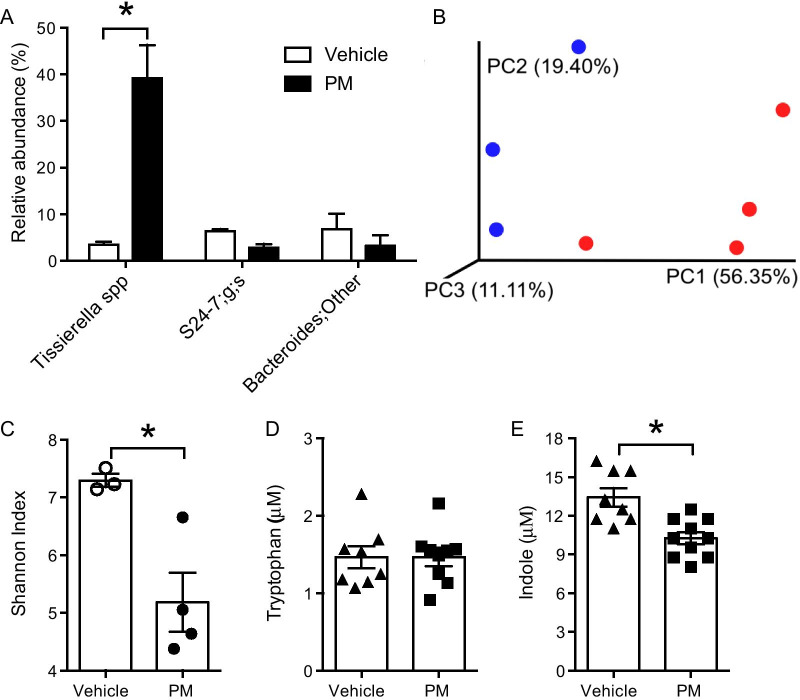
PM exposure induced dysbiosis of the lung microbiota and decreased microbial-derived indole in the lung. Neonatal mice (age: 3 days) were exposed to PM or vehicle and BALF was isolated at 4 dpe. BALF samples were subjected to 16s DNA sequencing for microbiota analysis. **A** Relative abundance of bacterial species. The top 25 bacterial species were compared, but only bacteria in either group > 5% were plotted. n = 3–4. **B** β-diversity measured by Bray–Curtis dissimilarity and visualized by PCoA analysis. PM (red)- or vehicle (blue)-exposed mice. n = 3–4. **C** α-diversity expressed as Shannon Index. n = 3–4. BALF samples were also analyzed for **D** tryptophan and **E** indole levels. n = 8–10, *p < 0.05

Lung microbes metabolize various endogenous components in the body and produce metabolites that activate signaling pathways controlling biological functions. Many of these metabolites are ligands for AhR, thus important for AhR activation and consequent IL22 production. We determined the effect of PM exposure on lung microbiota (4 dpe, no infection). Relative abundance of various bacterial species were determined, but only the species showing  > 5% difference were graphed. The data indicated significantly increased relative abundance of *Tissierella* species in lungs of PM-exposed mice (Fig. 3A). The beta-diversity, analyzed using Bray–Curtis dissimilarity index and represented using Principal Coordinates Analysis, showed that the microbiota of PM-exposed mice (red dots) was dissimilar to that of vehicle-exposed mice (blue dots) (Fig. 3B). Further, the alpha-diversity, measured by Shannon index analysis, significantly reduced in lungs of mice exposed to PM as compared to that of mice exposed to vehicle. (Fig. 3C). These data indicate that prolonged exposure to PM alters the lung microbiota.

We further measured the concentrations of tryptophan and indole (a tryptophan metabolite produced by resident microorganisms) in BALF from PM exposed mice at 4 dpe. There was no difference in the levels of tryptophan between vehicle and PM exposed mice (Fig. 3D). On the other hand, exposure to PM reduced the levels of indole in the lungs as compared to that in lungs of mice exposed to vehicle (Fig. 3E). Together, these data indicate that exposure to PM alters the lung microbiota leading to reduced production of microbial metabolites that act as AhR ligands.

### Treatment with rIL22 protects against PM exposure exacerbated lung injury and improves survival in influenza-infected mice

**Fig. 4:**
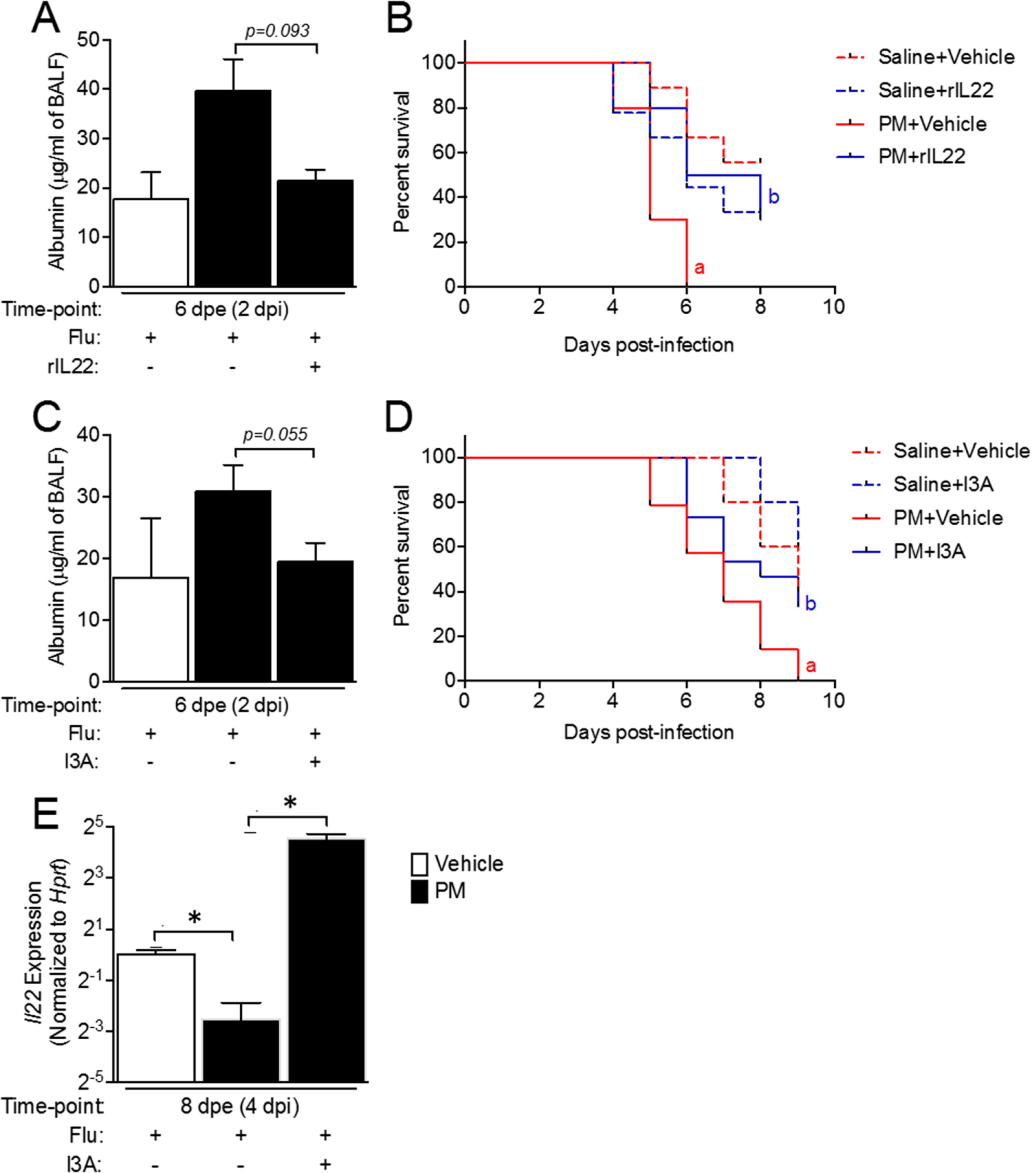
Treatment with rIL22 and I3A protects against PM exposure exacerbated lung epithelial damage and mortality in influenza infected mice. Neonatal mice were exposed to aerosolized PM and infected with influenza or sham infected at 4 dpe. Mice were treated with rIL22 (5 ng/g body weight) or I3A (5 µg/g body weight) delivered intranasally starting at 1 dpe, once a day. BALF was collected at 2 dpi and albumin concentration in BALF was measured using ELISA (**A**, **C**). Survival rate was calculated in mice treated with rIL22 **(B)** and I3A **(D)**. Lungs were isolated at 4 dpi from I3A treated mice and Il22 expression in lungs was assessed using qRT-PCR **(E)**. n = 3–5/group for BALF albumin, n = 3–4/group for lung Il22 expression, n = 5–9/group for survival. a *p* < 0.05, compared to Saline + Vehicle group, b *p* < 0.05, compared to Saline + rIL22 or Saline + I3A group

Previously, we reported that long-term exposure to PM causes lung epithelial damage and increases severity of influenza infection. Since IL22 expression was suppressed following influenza infection in PM exposed mice, we determined whether treatment with rIL22 could protect against PM-exacerbated lung injury in influenza-infected mice. The lung injury was assessed by measuring albumin concentration in BALF, as a marker of lung epithelial leakage, in PM-exposed influenza-infected mice treated with rIL22 or vehicle. As expected, exposure to PM increased the levels of albumin in the BALF as compared to that in vehicle-exposed mice. PM-exposed mice treated with rIL22 had reduced levels of albumin as compared to PM-exposed mice treated with vehicle (Fig. 4A). A separate set of mice, exposed to vehicle or PM and treated with rIL22 or vehicle, were observed for survival following influenza infection. PM-exposed mice showed significant reduction in survival compared to vehicle-exposed mice. Treatment with rIL22 significantly improved the survival in PM-exposed mice (Fig. 4B). These data suggest an important protective role of IL22 against PM-exacerbated lung injury and mortality in influenza-infected mice.

### Treatment with microbial metabolite I3A protects against PM-exacerbated lung injury and mortality in influenza-infected mice

PM exposure induced alteration of lung microbiota and lead to reduced microbial metabolites (some of which act as AhR ligands) in the lungs. To determine whether restoration of metabolites can protect against PM-induced severity of influenza infection, we treated the PM-exposed mice with indole-3-carboxaldehyde (I3A), a tryptophan metabolite produced by microbes. Treatment with I3A reduced the PM-exacerbated lung injury in influenza-infected mice, as indicated by reduced concentration of albumin in BALF (Fig. 4C), which was comparable to the concentrations observed in vehicle-exposed mice. In addition, PM-exposed and influenza-infected mice treated with I3A showed reduced mortality (Fig. 4D) as compared to PM-exposed mice treated with the vehicle. To further determine if I3A treatment can increase IL22, we measured the expression of IL22 in the lungs of I3A treated mice. Treatment with I3A significantly increased the expression of IL22 in lungs of PM-exposed mice at 4 dpi (Fig. 4E), which correlated with improved survival of these mice. Together, these data indicate that restoration of lung microbial metabolites provides resistance to PM-exacerbated influenza infection by increasing IL22 expression.

## Discussion

PM pollution is a major health hazard that affects people staying in industrial cities as well as countryside areas prone to wildfires and those that house various thermal power plants and hazardous waste sites [46–49]. There are several studies that associate PM exposure to increased hospitalizations, morbidity and mortality in infants and young children due to respiratory tract infections [10, 22–24]. However, there are very few studies reporting the underlying mechanisms for this. In the current study, we report the effects of PM exposure on lung IL22 levels and the lung microbiome, which contribute to an increased severity of influenza infection in infant mice. Continuous exposure to PM altered the lung microbiome leading to insufficient levels of tryptophan metabolites, such as indole and indole-3-carboxaldehyde, which are AhR ligands. Failure to induce expression of IL22 following influenza infection was associated with inefficient activation of AhR. Treatment with indole-3-carboxaldehyde or rIL22 protected against PM exacerbated lung injury and mortality following influenza infection. Overall, these data suggest an important mechanism of PM-exposure associated severe respiratory infections in infants and children.

Exposure to PM during early stages of life induces epithelial injury and airway remodeling [50, 51]. PM exposed infant mice, when infected with influenza virus, show increased severity of Flu infection. IL22 plays an important role in epithelial repair following injury aiding in epithelial cell proliferation inducing anti-apoptotic peptides and cytokines, reducing inflammation, and, at least against RSV, demonstrating anti-viral activity [27, 28, 52–54]. In addition, data suggest that IL22 is required to restrict the lung inflammation and bacterial secondary infection after influenza infection [34]. Our data demonstrate that exposure to PM initially induced the expression of IL22 in the lungs, however, with continuous exposure, IL22 expression was not maintained. Importantly, IL22 expression failed to increase following Flu infection in PM exposed mice. IL22 has been shown by some to be necessary for epithelial repair following influenza infection [52]. Upstream regulators of IL22 include IL23 and AhR [53]. We observed expression kinetics similar to IL22 for both IL23 and Cyp1a1 (a marker for AhR activation). These results collectively demonstrate that following PM exposure in neonatal mice, IL22 expression is attenuated which can exacerbate lung injury during subsequent respiratory tract infection.

We further investigated the cellular source of IL22 during neonatal PM exposure. It is well established that different innate and adaptive lymphoid cells produce IL22 [54, 55]. Recent studies have demonstrated that group 3 innate lymphoid cells (ILC3) and specifically retinoic acid-related orphan receptor γt (RORγt) + ILC3, are a major source of IL22 in the gut/lungs [56–58]. In the lung, IL22 producing ILC3 protect against allergic airway disease and *Streptococcus pneumoniae* infection [58]. Based on this, we investigated lung ILC3 cell levels during long-term PM exposure. Indeed, immediately following PM exposure in neonatal mice, we observed an increase in RORγt^+^ILC3 and IL22. The initial transient increase however was absent after 4 days of PM exposure and following Flu infection. ILC3 and IL22 increased only in vehicle but not in PM exposed mice. The timing of ILC3 and IL22 suggest a correlation between IL22 expression in lung and number of ILC3 cells.

In addition to IL23, several other factors are responsible for regulating IL22 expression and are capable of modulating the immune response to Flu following PM exposure. The lung resident microbiome, for example, is known to play a major role in tissue homeostasis and provides resistance against invading pathogens [59]. Tissue resident microbes produce various metabolites that mediate cellular processes for tissue homeostasis [40]. Indole and indole derivatives like indole-3-carboxaldehyde produced by microbes from catabolic degradation of tryptophan are AhR ligands and have been shown to induce IL22 expression [41, 42]. Our data show that exposure to PM lead to dysbiosis of lung microbiota, as indicated by reduced α-diversity (i.e. reduced numbers of total microbial species) and altered relative abundance within species. Alterations in resident microbiome can lead to varying levels of metabolites and affect biological activities. We saw reduced levels of indole and I3A in PM exposed mouse lungs. Different microbial species differ in their capabilities in breaking down substrates and metabolite production. Our results did not show any changes in *Lactobacillus* spp. (data not shown) which are major producers of indole and its derivatives from tryptophan. However, we saw a significant increase in *Tissierella* spp., which lack tryptophanase enzymes and, hence, cannot catabolize tryptophan into indole derived derivatives. In summary, this data indicates that changes in microbiota following PM exposure led to the decrease in AhR agonist (I3A), the absence of this agonist led to an inability to activate AhR as evidenced by the failure to activate Cyp1a1 transcription (a marker for AhR activation). The lack of AhR activation resulted in an inability to transcribe IL22 mRNA and thus make protein, and ultimately the failure to repair the epithelium damaged by exposure to PM. Thus, it is possible that the lack of these microbes led to a reduction in metabolites such as indole and I3A resulting in impaired or reduced IL22 expression.

To explore this, we investigated the effect of treatment with I3A or rIL22. After treatment with rIL22, we observed a significant increase in survival of the PM exposed and influenza infected mice. BALF albumin level were comparable to that in vehicle exposed mice, indicating the important role of IL22 in protection against the lung injury during PM exposure. These data further demonstrate the importance of IL22 throughout PM exposure and subsequent Flu infection for protection. Similarly, after treatment with I3A, a tryptophan metabolite, we saw reduced lung injury and mortality in PM exposed and influenza infected mice. Interestingly, we found an increase in IL22 expression in I3A treated mouse lungs, suggesting an important role of this microbial metabolite in maintaining and repairing the lung through the upregulation of IL22.

There are a few limitations to our study. First, the role of sex in this response to PM is unclear from our microbiome studies due to the fact that we had to pool samples. Second, our exposures in neonatal mice, while designed to mimic exposures in human infants, most likely fail to recapitulate the totality of exposures of a human infant. For example, infants are probably exposed in utero and throughout life at some low level with episodic increases in PM that are variable; whereas here we are only exposing neonatal mice and they are exposed on a daily basis to the same dose. Finally, while our data demonstrate that a microbial metabolite can alter IL22 levels and improve survival and epithelial integrity following influenza infection of PM exposed neonatal mice, beyond analyzing for changes in the microbiome we did not explore or try to identify the specific bacterial species responsible for the change in I3A following PM exposure.

## Conclusions

Collectively the findings from our animal studies demonstrate that early life exposure to PM results in the inability to induce IL22 activation upon Flu infection resulting in enhanced lung injury and Flu morbidity and mortality. We posit further that PM induced changes in the lung microbiome result in dysbiosis that leads to an inability to produce tryptophan metabolites critical for AhR and subsequent IL22 activation as treatment with I3A or rIL22 prevented lung injury and decreased mortality in Flu infected mice exposed to PM.

## Methods

### Animals

C57BL/6 breeders were obtained from Envigo (Indianapolis, IN) and time-mated to maximize birth cohorts. The sex of neonatal mice was not determined, and both male and female neonates were included in the experiments. For each experimental end point, a different cohort of mice was used. All animals were housed in ventilated cages with food and water provided ad libitum, under controlled conditions with 12-h light/dark cycle, humidity and temperature. All animal protocols were prepared, and experiments were followed in accordance with Guide for the Care and Use of Laboratory Animals and were approved by Louisiana State University Institutional Animal Care and Use Committee.

### Neonatal exposure to combustion-derived particulate matter and infection

Combustion-derived particulate matter was generated by our colleague Dr. Lavrent Khachatryan and Dr. Slawo Lomnicki (LSU) as previously described [60]. PM containing EPFRs (DCB230) was dissolved in 25 mL of saline and 0.02% Tween-80 and dispersed with a probe sonicator. The final concentration of the PM solution was 0.2 mg/mL. Neonatal mice (3 days of age) were placed in a whole-body exposure chamber and exposed to nebulized particles for 30 min per day as previously described until the respective experiment endpoints [61]. The average daily exposure concentration was 200 μg/m^3^. Neonatal pups were infected with 1.25 TCID_50_/neonate of mouse adapted human influenza A strain A/PR/8/34 (H1N1) at 4 dpe (Advanced Biotechnologies, Inc) in 10 μL of Dulbecco’s phosphate-buffered saline (DPBS). Vehicle-exposed mice were dosed with 10 μL of DPBS. A timeline of exposures and treatments is presented in the Fig. 1D.

### Collection of BALF

Bronchoalveolar lavage fluid (BALF) was collected by instilling PBS into the lungs intratracheally and aspirating with a syringe. Collected BALF was centrifuged; the supernatant was collected and stored at − 80 °C until 16S sequencing and metabolite analysis.

### Lung cell isolation and flow cytometry

Mice were humanely euthanized and retrograde lung perfusion via the right ventricle of the heart with isotonic PBS was used to flush the excess blood. Lungs were then excised and collected in Hank’s Balanced Salt Solution (HBSS) (HyClone, UT) on ice. A single-lung suspension was generated as described previously [25]. Briefly, the individual lungs were cut into small pieces and collected in 2.4 ml of HBSS buffer containing 1 mg/ml collagenase I (Invitrogen, MA), and 150 μg/ml DNase I (Sigma-Aldrich, MO). Further the lungs were mechanically dissociated with the gentleMACS™ Octo Dissociator (Miltenyi Biotech, Germany) and incubated with continuous shaking (200 rpm) at 37 °C for 30 min. Following 30 min incubation, the lungs were dissociated a second time in Octo Dissociator before being passed through a 40 μm cell strainer to obtain single cell suspension. The resulting single cell suspension was treated with RBC lysis buffer (BioLegend, San Diego, CA) to lyse the residual RBCs according to manufacturer's protocol. ILC3 subset was identified as Lin^−^, CD45^+^, CD90.2^+^, CD127^+^ and RORyt^+^ [62, 63].The antibodies used in the study for staining were Alexa Fluor 700-CD45 (clone-30-F11; BD Biosciences, CA), FITC-CD90.2 (clone- 30-H12; BD Biosciences, CA), PE-Cy7-CD127 (clone-A7R34; Tonbo biosciences, CA), APC-RORγt (clone-B2D; eBiosciences, CA) and PE-IL22 (clone-1H8PWSR; eBiosciences, CA). Fixable live/dead marker BV510 (Tonbo Biosciences, San Diego, CA) was used to exclude dead cells from analysis. Following antibody staining, flow cytometry was performed on a Canto II flow cytometer (BD Biosciences) and data was analyzed with FlowJo software (v10 for Windows; Tree Star; Ashland, OR, USA).

### Gene expression

RNA was extracted from individual mouse lungs using the RNeasy Plus Universal Kit (Qiagen, Germantown, MD). The Superscript III First Strand Synthesis Kit (Thermo Fisher, Waltham, MA) was used to reverse transcribe 2 μg of RNA from each mouse into cDNA. Real time PCR for IL22, IL23, and Cyp1a1 was performed on the LightCycler 480 real time PCR thermal cycler (Roche Diagnostics, Indianapolis, IN). Data was normalized to Hprt and plotted as relative gene expression.

### Assays

Assays for Tryptophan, indole (Sigma Aldrich, St. Louis, MO) and albumin (Sigma Aldrich, St. Louis, MO) levels were performed using the commercially available kits according to manufacturer's protocol.

### 16s sequencing and bioinformatic analysis

BALF was collected 4 h after the last exposure and DNA was isolated using kit from Qiagen (Germantown, MD) according to manufacturer’s protocol. Samples were sent to Center for Clinical and Translational Science, University of Alabama at Birmingham for 16S sequencing. Hypervariable V4 regions of the bacterial 16S rRNA gene was amplified from each sample using the unique barcoded primers. Further the amplicon libraries were then purified from the gel using QIAquick Gel Extraction kit (Qiagen, Germantown, MA). Finally, after the purification process next generation sequencing was performed on the libraries on Illumina MiSeq system using 250 bp paired end reads according to the protocol published by Kumar et al. [64]. QIIME 2 version 2018.4.0 (RRID: SCR_008249) was used for processing and analysis of sequence data having a minimum length of 250 bp. For demultiplexing Qiime 2 demux was used and quality control was done using deblur quality control method. Subsequently, alpha diversity was measured using Chao1 index and Shannon Index in Qiime2. Beta diversity and the community similarity was measured via unweighted and weighted unifrac distance matrix. To identify significant features between groups PERMANOVA with 999 permutations was used. The data was normalized in Calypso using cumulative sum scaling normalization for multivariate tests. To address the false discovery rate (FDR) two-way repeated measures ANOVA in conjunction with Benjamini–Hochberg multiple-inference correction with Dunnett’s correction was used to test for significant differences in alpha diversity and differential taxa abundance between groups. Significance was defined as *p* < 0.05 after FDR adjustment (Prism v8.0, GraphPad Software, San Diego, CA) (Calypso).

### Treatment with rIL22 and indole-3-carboxaldehyde (I3A)

To understand the effect of IL22 and I3A in PM induced exacerbation of flu severity, air exposed wild type neonatal mice were treated with rIL22 (Sigma-Aldrich, St. Louis, MO) and I3A (PeproTech, Inc., East Windsor, NJ). The neonatal mice were anesthetized using isoflurane and rIL22 (5 ng/g body weight) and I3A (5 µg/g body weight) were administered intranasally once a day starting from 1 dpe. At 4 dpe and 4 h after administration of rIL22 or I3A, mice were once again anesthetized with isoflurane and then infected with influenza (1.25 TCID_50_ per mouse) or sham infected. Lungs were isolated at 4 dpi from the treated mice.

### Statistical analysis

Statistical analysis and figure generation were performed on GraphPad Prism statistical software (Prism v8.0, GraphPad Software, San Diego, CA) and results are expressed as ± SEM. One-way ANOVA or Two-way ANOVA with Mann-Whitey test and unpaired t-tests were performed to determine the difference between the groups. p values less than 0.05 were considered statistically significant.

## Data Availability

The datasets used and/or analyzed during the current study are available from the corresponding author on reasonable request.
